# A Self-Assembled Electro-Active M_8_L_4_ Cage Based on Tetrathiafulvalene Ligands

**DOI:** 10.3390/ma7010611

**Published:** 2014-01-22

**Authors:** Sébastien Goeb, Sébastien Bivaud, Vincent Croué, Vaishali Vajpayee, Magali Allain, Marc Sallé

**Affiliations:** LUNAM Université, Université d’Angers, CNRS UMR 6200, Laboratoire MOLTECH-Anjou, 2 bd Lavoisier, 49045 Angers Cedex, France; E-Mails: sebastien.bivaud@etud.univ-angers.fr (S.B.); vincent.croue@etud.univ-angers.fr (V.C.); vaishali.vajpayee@univ-angers.fr (V.V.); magali.allain@univ-angers.fr (M.A.)

**Keywords:** self-assembly, metalla-cages, metal-driven, tetrathiafulvalene, redox

## Abstract

Two self-assembled redox-active cages are presented. They are obtained by coordination-driven self-assembly of a tetra-pyridile tetrathiafulvalene ligand with *cis*-M(dppf)(OTf)_2_ (M = Pd or Pt; dppf = 1,1′-bis(diphenylphosphino)ferrocene; OTf = trifluoromethane-sulfonate) complexes. Both species are fully characterized and are constituted of 12 electro-active subunits that can be reversibly oxidized.

## 1. Introduction

The coordination-driven self-assembly methodology has proven during the last decades to be very efficient for the preparation of discrete polygons (2D) or polyhedra (3D) otherwise challenging to synthesize through conventional covalent multi-step synthesis [1–13]. This approach involves a coordination process between a rigid organic ligand and a metal center of complementary geometry, to provide thermodynamically stable discrete assemblies in high yields. In particular, nitrogen-based binding sites such as substituted pyridines are often used in presence of square planar Palladium (II) or Platinum (II) salts.

The corresponding host cavities offer new promising opportunities for applications in molecular recognition or even in guest transport [14–16]. In this context, very few examples of coordination-driven redox-active self-assembled discrete structures have been described so far. Their constituting ligands are usually based on electro-deficient skeletons such as triazine [17,18] or perylene diimide [19–25] units and more rarely on oxidable fragments [26,27]. It has to be noted that the electro-activity can also be located on the metal complex [28–38] or on pendant units [39–43] instead of being centered on the side-walls. In the course of our studies related to the preparation of electron-rich functional metallosupramolecular discrete architectures, we recently described the first metalla-cycles [44,45] and metalla-cages [46,47] constructed from derivatives of the π-donating tetrathiafulvelene unit [*i.e.*, bispyrrolo(tetrathiafulvalene) (BPTTF) or the so-called extended-tetrathiafulvalene (*ex*TTF)]. The π-donating ability of tetrathiafulvalene (TTF) derivatives is well established and is responsible for the success of this redox-unit which is used in various molecular and supramolecular switchable systems [48–51].

Herein, we present an original example of coordination-driven self-assembled architectures involving the parent TTF redox-active framework and *cis*-blocked Pd(II) and Pt(II) salts.

## 2. Results and Discussion

### 2.1. Ligand **L1**: Synthesis and Characterization

**Scheme 1.**
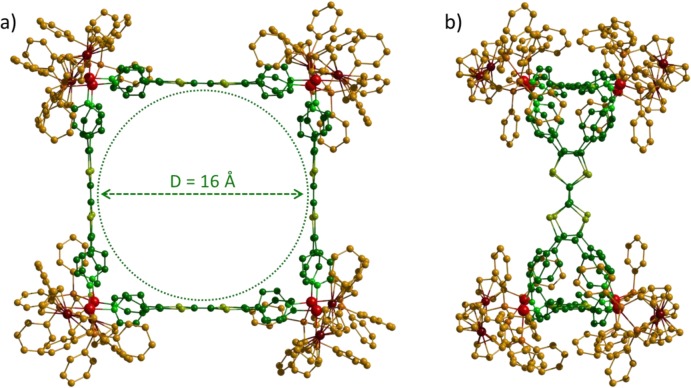
Synthesis of ligand **L1**: 4-Iodopyrine (5 equiv.), Pd(OAc)_2_ (0.25 equiv.), P(tBu)_3_·HBF_4_ (0.75 equiv.) Cs_2_CO_3_ (5 equiv.), Dioxane, reflux, 24 h, 71%.

New ligand **L1** was obtained in one step from the pristine TTF through a Palladium-catalyzed C–H cross-coupling reaction [52,53] with 4-iodopyrine (Scheme 1). This reaction is run in good yields (71%) when taking into account that four C–C bonds are simultaneously formed.

**Figure 1.**
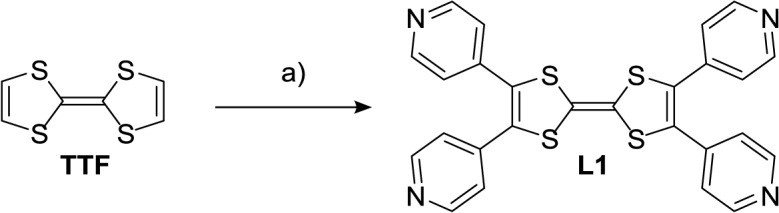
X-Ray structure of ligand **L1**: (**a**) side view and (**b**) top view.

Single crystals of ligand **L1** were obtained by slow diffusion of hexane in a solution of **L1** in dichloromethane. The XRD analysis (Tables S1) revealed that the TTF skeleton and the four nitrogen atoms are coplanar (Figure 1a) and set in a rectangle defined by a length of 13.1 Å and a width of 6.7 Å (Figure 1b). The electro-deficient pyridile plans are highly rotated related to the TTF backbone (*i.e.*, between 31° and 77°) and are therefore poorly conjugated to the donating part of the molecule. Consequently, the π-donating ability of ligand **L1** should be only moderately altered compared to the parent TTF.

### 2.2. Assemblies **1** and **2**: Synthesis and Characterization

**Figure 2.**
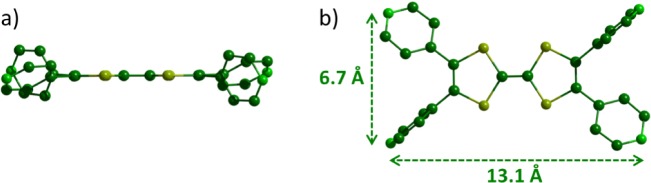
^1^H NMR (CD_3_NO_2_): (**a**) **L1** (*); (**b**) *cis*-Pt(dppf)(OTf)_2_; (**c**) **2**; (**d**) **1**; and (**e**) DOSY NMR of **1**. (*) ^1^H NMR of ligand **L1** was recorded in a mixture of CD_3_NO_2_/CDCl_3_ (2/1).

Ligand **L1** was engaged in a self-assembly process with *cis*-M(dppf)(OTf)_2_ (M = Pt or Pd) complexes in nitromethane at 50 °C and the reaction was monitored by ^1^H NMR (Figures 2, S1). Several discrete architectures are potentially expected from this reaction, according to the number of ligand **L1** involved in the final structures. In both cases, the reaction converged to one unique species **1** (Pt complex) or **2** (Pd complex) in 2 h and 5 min respectively. The self-assembled discrete structures could be isolated by precipitation from Et_2_O. Compared to ligand **L1** (Figure 2a) and starting metal complexes (Figure 2b), assemblies **1** (Figure 2d) and **2** (Figure 2c) present pyridile signals which are upfield-shifted (Hα ≈ 8.5 ppm and Hβ ≈ 7.1 ppm) and one singlet for each proton corresponding to the cyclopentadienyl units (≈4.8 ppm). This behavior is expected and has already been reported for similar compounds [46]. One singlet is observed for each of the ^19^F and ^31^P NMR spectra of **1** and **2** (Figures S4, S5, S9, S10), and the corresponding DOSY NMR spectra exhibit a single alignment of signals (Figures 2e, S11). All these data are in agreement with the formation of a unique species during the self-assembly reaction.

Remarkably, both self-assemblies present the same diffusion coefficient in solution (D ≈ 2.2 × 10^−10^ m^2^·s^−1^) extracted from the DOSY experiments. This result indicates that both species are of similar size with an estimated hydrodynamic radius of *ca.* 16.5 Å calculated from the Stokes-Einstein equation [54]. This value is in line with the formation of a large discrete self-assembled architecture but does not allow discriminating between a M_6_L_3_ or a M_8_L_4_ species.

**Figure 3.**
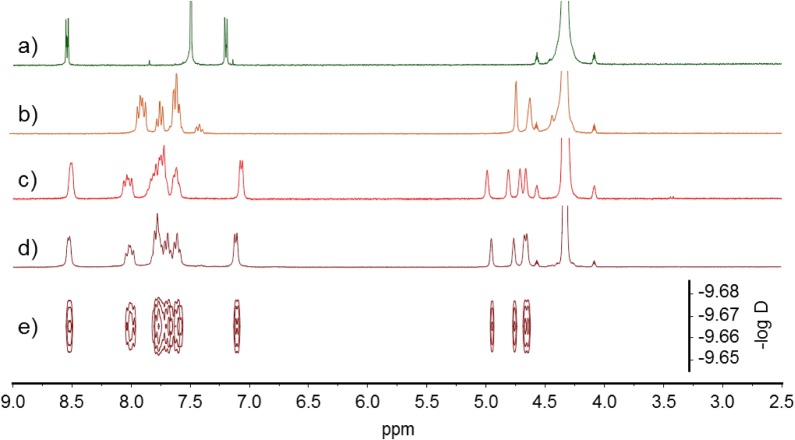
ESI-MS of self-assembly **1** in CH_2_Cl_2_ (C = 10^−3^ M).

High resolution ESI mass spectrometry experiments were carried out from dichloromethane solutions of **1** (Figure 3 and Figure S13) and **2** (Figure S14) and confirm the formation of M_8_L_4_ architectures with peaks corresponding to multi-charged species [M-3TfO^−^]^3+^ (m/z = 3327.6752 (**1**) and 3091.5087 (**2**)), [M-4TfO^−^]^4+^ (m/z = 2459.0238 (**1**) and 2280.8943 (**2**)) and [M-5TfO^−^]^5+^ (m/z = 1937.0275 (**1**)), as well as a good matching between the experimental and theoretical isotopic patterns.

**Figure 4.**
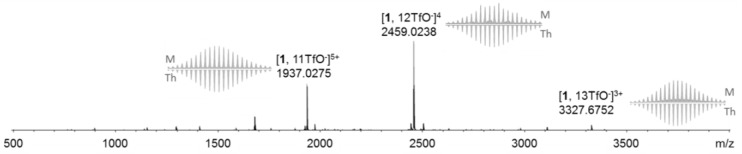
Molecular force field (MM+) model of square **1**: (**a**) front view and (**b**) side view.

**Scheme 2.**
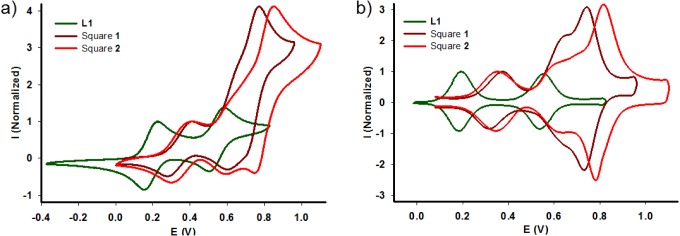
Synthesis of polygons **1** and **2**: (**A**) *cis*-M(dppf)(OTf)_2_ (2 equiv.), nitromethane, 50 °C; for **1**: M = Pt, 2 h, 91%; for **2**: M = Pd, 5 min, 87% and (**B**) Hypothetical alternative structure

Depending on the orientation of the ligands inside the structure, two types of geometry are conceivable for the M_4_L_8_ assemblies (*i.e.*, the rectangular ligands are connected to the metal centers through their short (Scheme 2A) or long side (Scheme 2B). Molecular force field (MM+) studies were undertaken to determinate the most probable structure. Geometry optimization could not yield a symmetric stationary point for the B assembly (Scheme 2B) due to a high structural strain. In contrast, a highly symmetric assembly could be reached for the geometry A (Figure 4). As expected, the structural characteristics of the tetra-pyridyl ligand within the metalla-assemblies are close to those of free **L1** (Figure 1) and a distance of 16 Å is found between two facing TTF plans. A total diameter of 36 Å was found for complex **1** in reasonable accordance with the value determined from the DOSY NMR experiment.

### 2.3. Redox Properties

**Figure 5.**
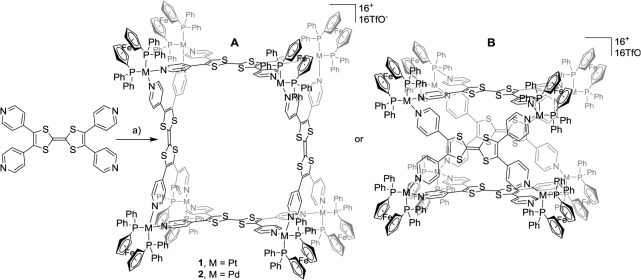
(**a**) Cyclic voltammogram and (**b**) deconvoluted cyclic voltammogram of ligand **L1** (C = 10^−3^ M, CH_3_CN/CH_2_Cl_2_, 0.1 M *n*Bu_4_NPF_6_, 100 mV·s^−1^, Cgr) and of squares **1** and **2** (C = 5 × 10^−4^ M, CH_3_CN, 0.1 M *n*Bu_4_PF_6_, 20 mV·s^−1^, Cgr), V *vs* Fc/Fc^+^.

Redox properties of ligand **L1**, complexes **1** and **2** were studied by cyclic voltammetry (Figure 5). As usually observed with TTF derivatives, two reversible oxidation waves are observed for ligand **L1** and are assigned to the successive generation of the cation-radical and dication states. A lower π-donor ability is found compared to the parent unsubstituted TTF, with a first redox potential which is shifted by +230 mV. This behavior is ascribed to the presence of four electro-deficient pyridile units on the periphery of the TTF backbone. Three reversible redox processes are observed in the case of the self-assembled metalla-cages **1** and **2**. The first two are assigned to the TTF side walls and the third one to the corner ferrocene units. Interestingly, the latter can be used as an internal reference to address the number of electrons exchanged along the redox processes. The relative intensities collected from the deconvoluted cyclic voltammogram (Figure 5b) agree with a successive one, one, two electrons oxidation sequence. Since the assembly encompasses four TTF and eight ferrocene redox units, such observation indicates that full oxidation at a potential of 1.0 V *vs* Fc/Fc^+^ leads to the reversible generation of sixteen positive charges in addition to those centered on the eight M(II) complexes. Finally, it has to be mentioned that the CV shape remains unchanged upon several scanning between 0.0 V and 1.0 V *vs* Fc/Fc^+^. Nevertheless, a repeated cycling to the upper limit of potentials results in a progressive decreasing of the redox waves intensities, in particular with the Pd derivative, which we attribute to a passivation of the electrode. Therefore, this stability and the possibility which is offered by such systems to control the charge (up to sixteen) on the periphery of the cavity, open promising perspectives in terms of guest binding and transport.

## 3. Experimental Section

### 3.1. Chemicals

All reagents were of commercial reagent grade and were used without further purification. Complexes *cis*-Pd(dppf)(OTf)_2_ and *cis*Pt(dppf)(OTf)_2_ [55], (dppf = 1,1′-bis(diphenylphosphino) ferrocene; OTf = trifluoromethane-sulfonate) were synthesized as described in literature. Silica gel chromatography was performed with a SIGMA Aldrich Chemistry SiO_2_ (pore size 60 Å, 40–63 μm technical grades).

### 3.2. Instrumentation

The 300.3 (^1^H), 75.5 (^13^C), 121.6 (^31^P) and 282.6 MHz (^19^F) NMR spectra were recorded at room temperature using perdeuterated solvents as internal standards (^1^H), external H_3_PO_4_ solution (^31^P) and CFCl_3_ (^19^F), on a NMR Bruker Avance III 300 spectrometer (Bruker, Rheinstetten, Germany). MALDI-TOF-MS spectra were recorded on a MALDI-TOF Bruker Biflex III instrument using a positive-ion mode. ESI-MS spectra were achieved on a Bruker MicrO-Tof-Q 2 spectrometer in CH_2_Cl_2_. Cyclic voltammetry experiments were carried out on an ALS electrochemical analyzer model 660 and the conditions were the following: 0.1 M *n*Bu_4_NPF_6_ in acetonitrile or acetronitrile/methylene chloride (1/1 v/v), Ag/Ag^+^ reference electrode, GC or Pt working electrode, and Pt counter electrode, calibrated using internal ferrocene. Elemental analyses were achieved on a Thermo Electron analyzer.

### 3.3. Single-crystal X-ray Crystallography

X-ray single-crystal diffraction data were collected at low temperature on a Bruker KappaCCD diffractometer, equipped with a graphite monochromator utilizing MoKα radiation (λ = 0.71073 Å). The structure was solved by direct method and refined on F^2^ by full matrix least-squares techniques using SHELX97 (Programs for Crystal Structure Analysis (Release 97-2). Sheldrick, G.M. [56]) package. All non-hydrogen atoms were refined anisotropically. Absorption was corrected by SADABS program (Sheldrick, Bruker, 2008). The H atoms were found by Fourier difference map. CCDC reference number CCDC 951195 (**L1**) contains the supplementary crystallographic data for this paper. These data can be obtained free of charge from The Cambridge Crystallographic Data Centre [57].

### 3.4. Molecular Modeling

Molecular modeling was performed by using the molecular mechanics force field MM+ method from the HyperChem 8.0.3 program (Hypercube, Inc., Waterloo, ON, Canada,) configured *in vacuo*, with a RMS of 10^−5^ kcal/mole, a number of maximum cycles of 32500, and a Polak-Ribiere algorithm. Counter anions were omitted to simplify the calculation.

### 3.5. Experimental Procedure and Characterization Data

#### 3.5.1. 4,4′,5,5′-tetra(pyridin-4-yl)-2,2′-bi(1,3-dithiolylidene) (**L1**)

To a suspension of palladium acetate (82 mg, 0.36 mmol), tri-*tert*-butylphosphine tetrafluoroborate (320 mg, 1.10 mmol) and cesium carbonate (2.40 g, 7.30 mmol) stirred for 10 min at 90 °C under argon in distilled dioxane (20 mL) was added an argon degassed solution of tetrathiafulvalene (300 mg, 1.46 mmol) and 4-iodopyridine (1.50 g, 7.34 mmol) in dioxane (20 mL). The reaction was stirred under reflux for 24 h. After cooling, a large excess of dichloromethane and water were added. The aqueous phase was extracted and the organic extracts were washed with brine, dried over magnesium sulfate, filtered and concentrated. The residue was purified by chromatography on silica gel (deactivated with triethylamine 1%) eluting from dichloromethane to dichloromethane/methanol (97/3 v/v) to give a red powder (530 mg, 1.03 mmol, 71%). Crystals (dark red needles) were obtained by slow diffusion of hexanes in dichloromethane. Melting Point: >260 °C. ^1^H NMR [300 MHz, CDCl_3_]: δ (ppm) = 8.55 (dd, *^3^J =* 4.5 Hz, *^4^J = 1.6* Hz, 8H, Hα), 7.09 (dd, *^3^J =* 4.5 Hz, *^4^J = 1.6* Hz, 8H, Hβ). ^13^C NMR [75 MHz, CDCl_3_]: δ (ppm) = 150.6, 139.4, 129.6, 123.1, 109.0. Calculated [C_26_H_16_N_4_S_4_]: 512.69; Observed (MALDI-TOF): 512.5.

#### 3.5.2. Complex 1

A mixture of ligand **L1** (10.0 mg, 19.5 μmol) and *cis*-Pt(dppf)(OTf)_2_ (40.9 mg, 39.1 μmol) in anhydrous nitromethane (2 mL) was heated at 50 °C for 2 h under argon. After cooling, diethyl ether (10 mL) was added and the mixture was centrifuged. The residue was washed with diethyl ether and dried under vacuum to give complex **1** (46.3 mg, 4.4 μmol, 91%) as a dark orange solid. ^1^H NMR (CD_3_NO_2_) δ = 8.53 (d, *^3^J =* 5.9 Hz, 32 H), 8.01 (m, 32 H), 7.80–7.61 (m, 128 H), 7.12 (d, *^3^J =* 5.9 Hz, 32 H), 4.95 (brs, 16 H), 4.77 (brs, 16 H), 4.67 (m, 32 H); ^19^F NMR (CD_3_NO_2_) δ −81.30; ^31^P NMR (CD_3_NO_2_) δ = 1.64; ESI-MS m/z: 1937.0275 ([**1**-11OTf]^5+^), 2459.0238 ([**1**-12OTf]^4+^), 3327.6752 ([**1**-13OTf]^3+^); mp > 260 °C.

#### 3.5.3. Complex 2

A mixture of ligand **L1** (10.0 mg, 19.5 μmol) and *cis*-Pd(dppf)(OTf)_2_ (37.4 mg, 39.1 μmol) in anhydrous nitromethane (2 mL) was heated at 50 °C for 5 min under argon. After cooling, diethyl ether (10 mL) was added and the mixture was centrifuged. The residue was washed with diethyl ether and dried under vacuum to give complex **2** (41.2 mg, 4.2 μmol, 87%) as a dark red solid. ^1^H NMR (CD_3_NO_2_) δ = 8.51 (d, *^3^J =* 5.9 Hz, 16 H), 8.04 (m, 32 H), 7.81–7.62 (m, 128 H), 7.07 (d, *^3^J =* 5.9 Hz, 16 H), 4.99 (brs, 16 H), 4.81 (brs, 16 H), 4.71 (brs, 16 H), 4.67 (brs, 16 H); ^19^F NMR (CD_3_NO_2_) δ = 83.77; ^31^P NMR (CD_3_NO_2_) δ = 31.81; ESI-MS m/z: 2280.8943 ([**2**-12OTf]^4+^), 3091.5087 ([**2**-13OTf]^3+^); mp > 260 °C.

## 4. Conclusions

The synthesis and the characterization of two coordination-driven self-assembled cages is depicted. They are constructed from an electron-rich TTF based ligand **L1** and an electro-active *cis*-M(dppf)(OTf)_2_ (M = Pd or Pt) complex. They exhibit an internal cavity of 16 Å in diameter, which is surrounded by four redox-active TTF and eight ferrocene units. All of them can be oxidized, offering the possibility to reversibly generate up to sixteen positive charges on the assembly. This full control over the charge state of the cavity opens very promising perspectives, in particular for answering the key question of the triggering of the guest binding.

## Supplementary Materials

Supplementary File 1
