# Supplementary material for: A Self-Assembled Electro-Active M8L4 Cage Based on Tetrathiafulvalene Ligands
Source: Materials (Basel). 2014 Jan 22;7(1):611–22. doi: 10.3390/ma7010611 (PMC5453136; doi:10.3390/ma7010611)

Supplementary

NMR Spectra

Figure S1. ^{1}H NMR spectrum of **L1** in $CDCl_3$.

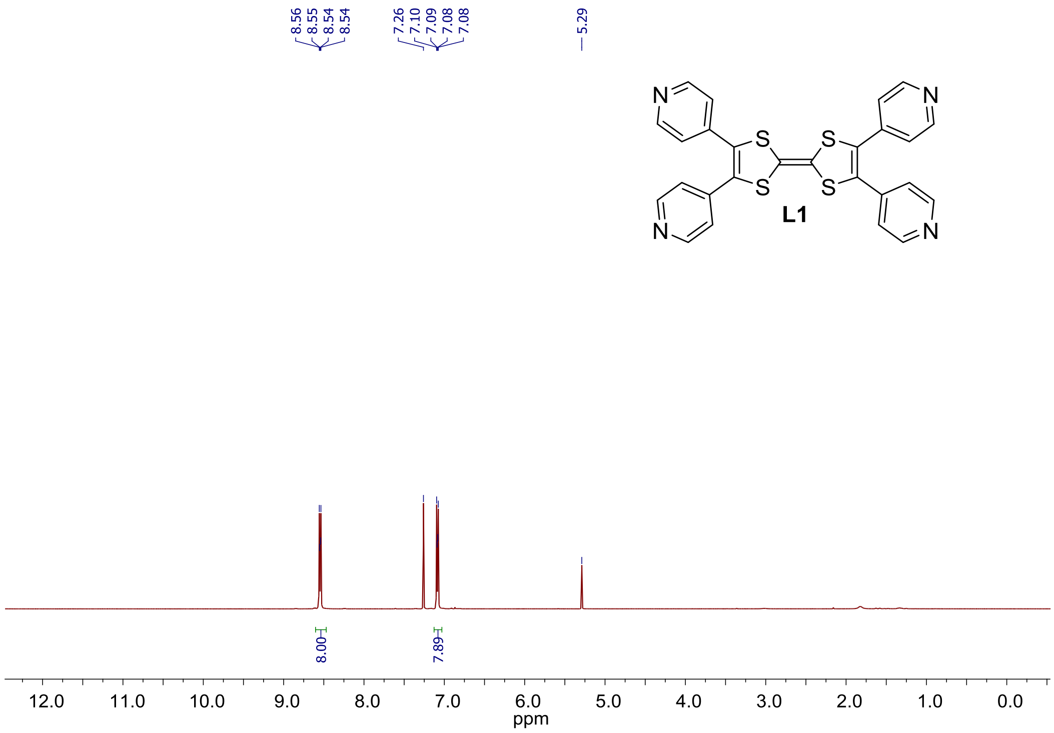

Figure S2. ^{1}H NMR spectrum of **L1** in $CDCl_3$.

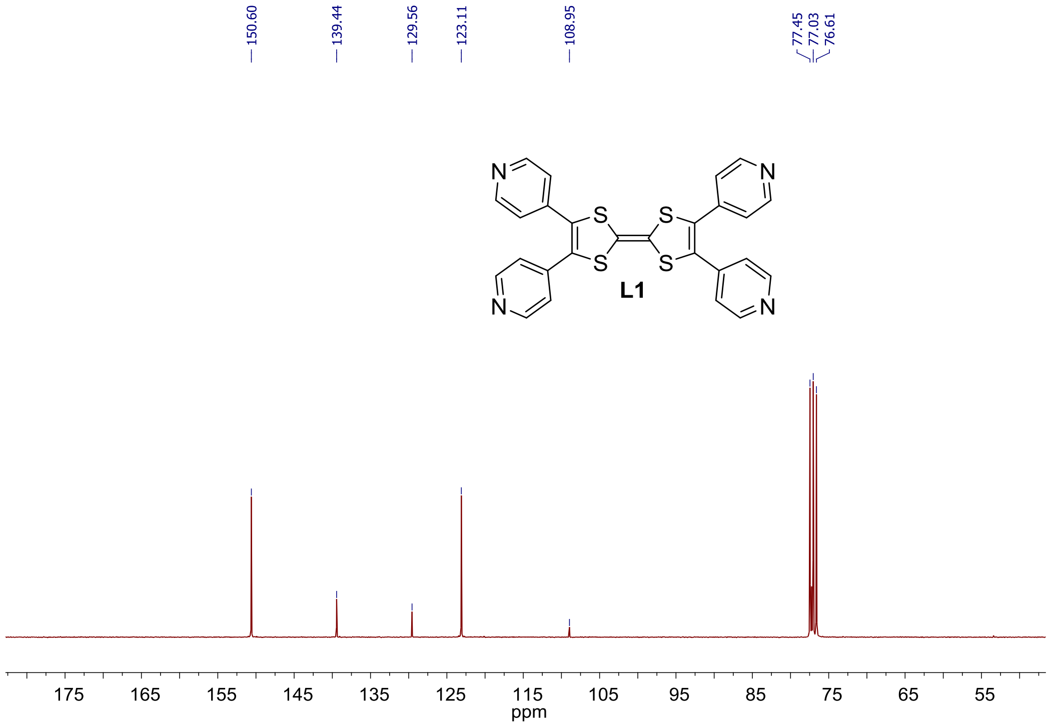

Figure S3. ^{1}H NMR spectrum of **1** in CD_3NO_2.

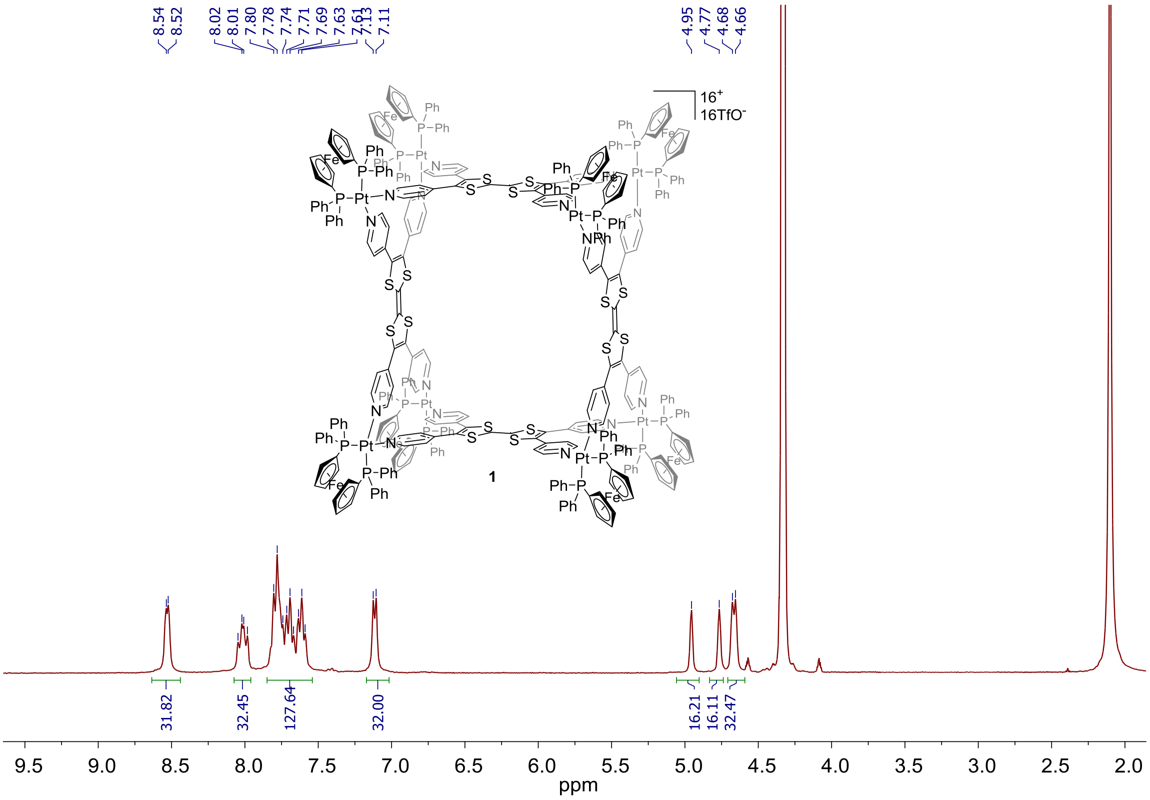

Figure S4. ^{19}F NMR spectrum of **1** in CD_3NO_2.

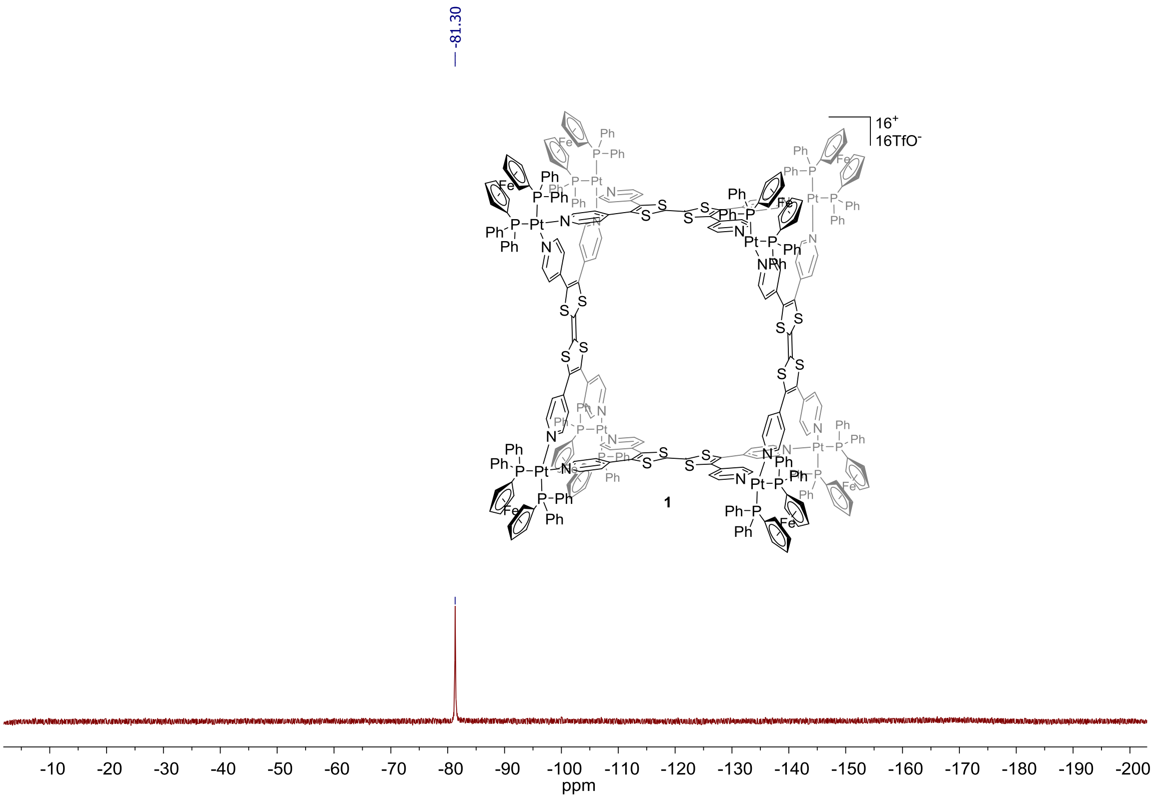

Figure S5. ^{31}P NMR spectrum of **1** in CD_3NO_2.

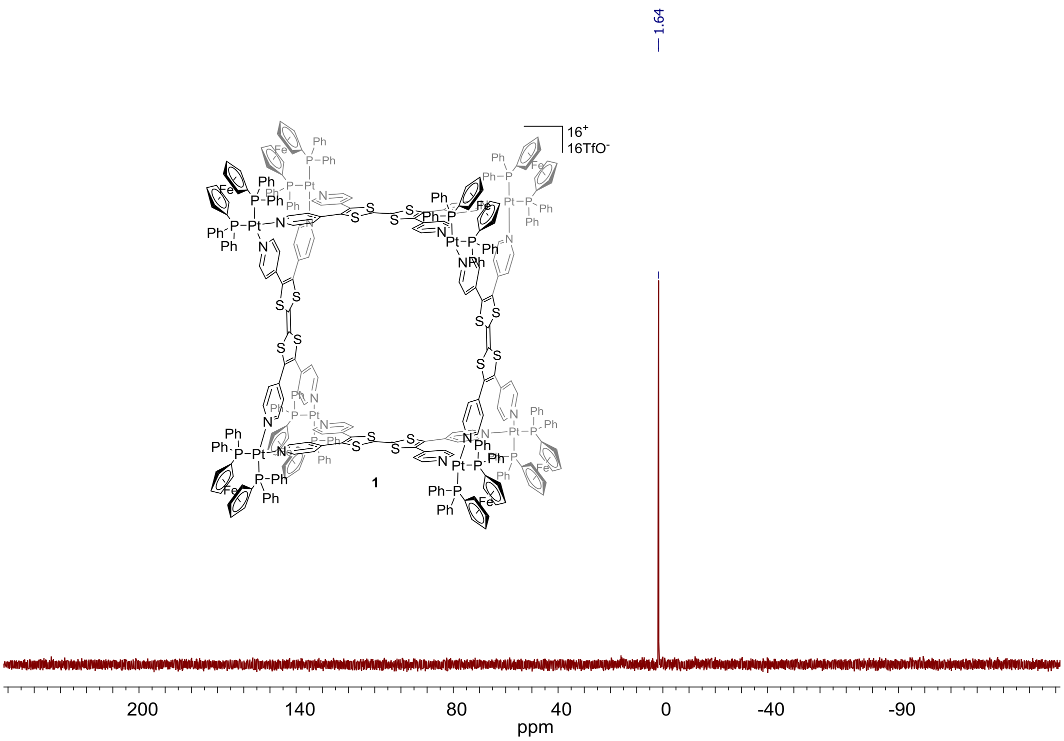

Figure S6. ^{1}H DOSY spectrum of **1** in CD_3NO_2.

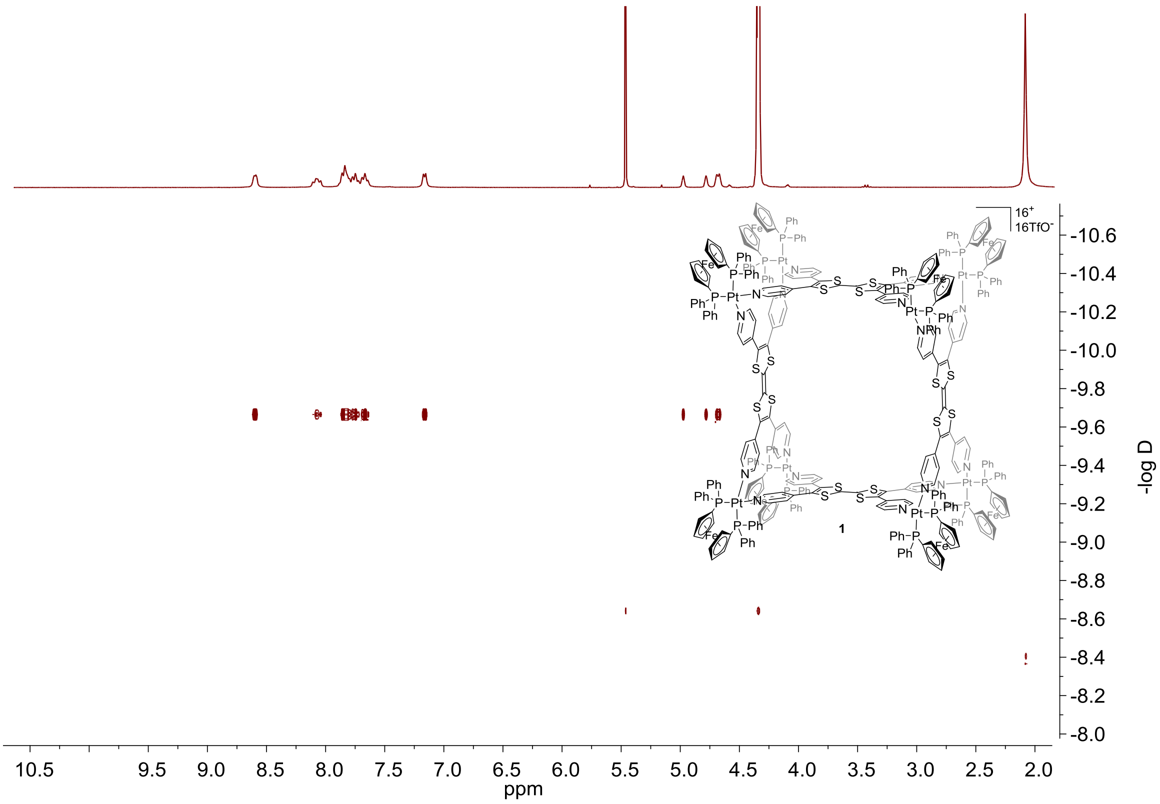

Figure S7. ^{1}H COSY spectrum of **1** in CD_3NO_2.

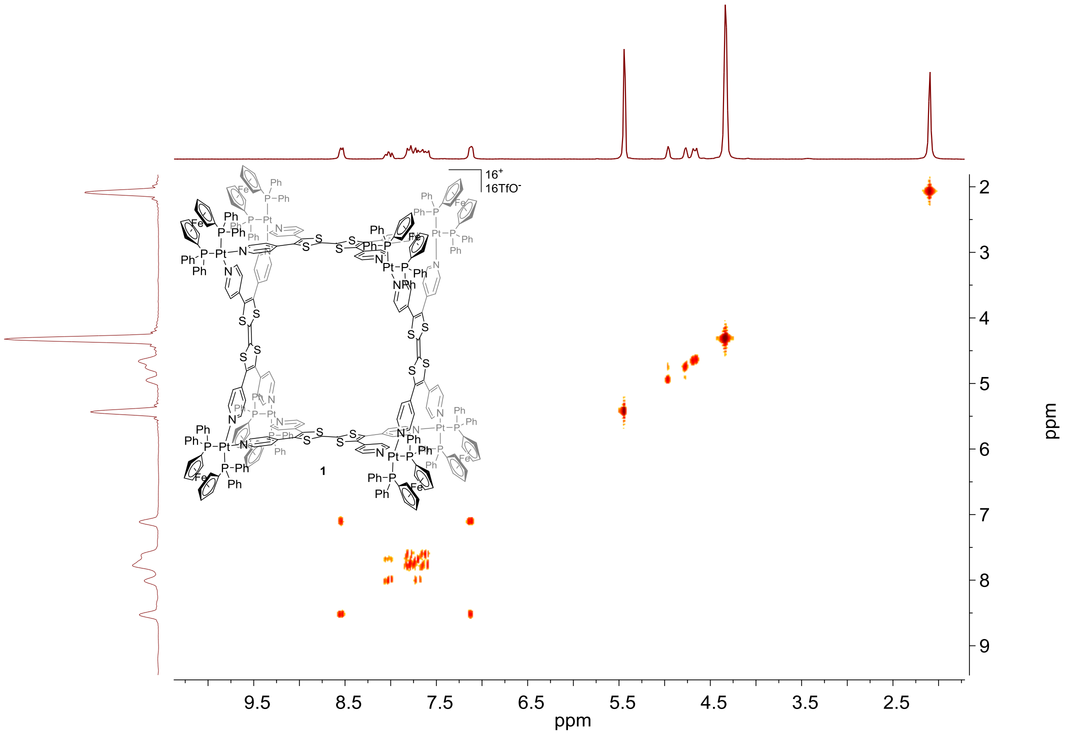

Figure S8. ^{1}H NMR spectrum of **2** in CD_3NO_2.

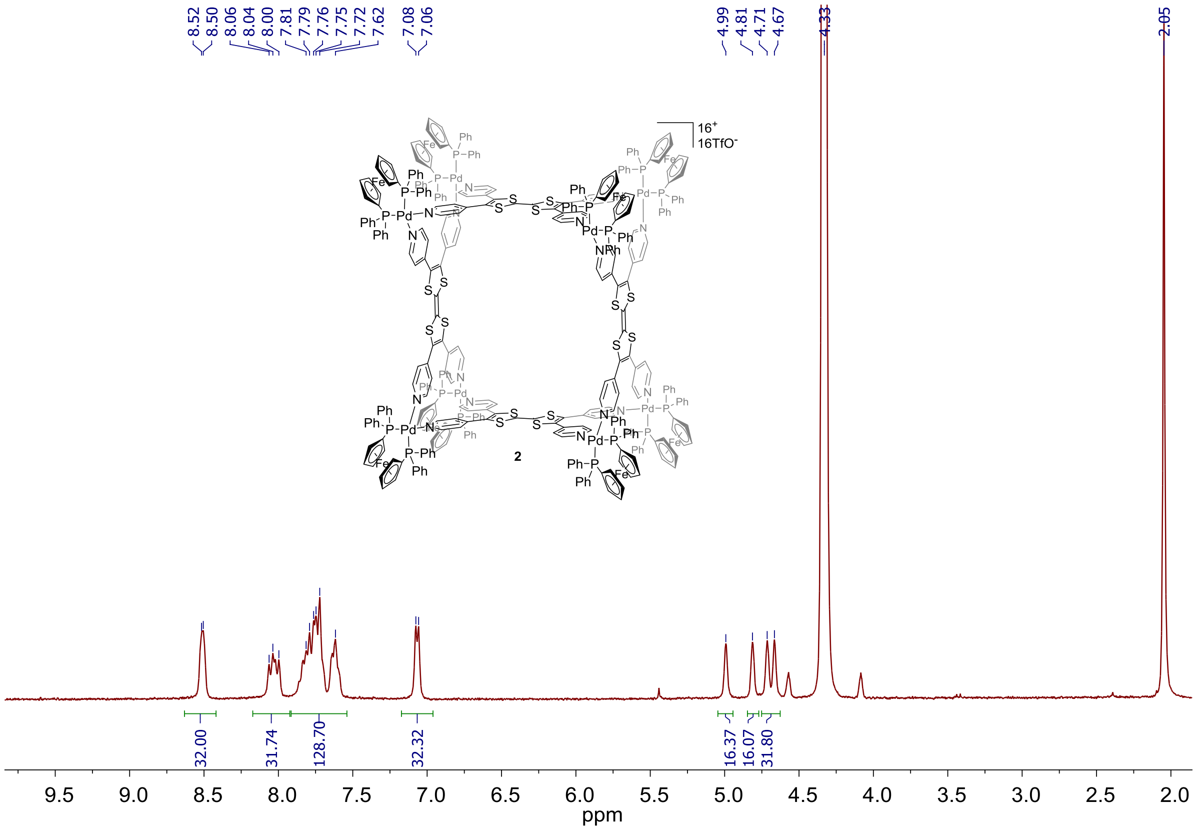

Figure S9. ^{19}F NMR spectrum of **2** in CD_3NO_2.

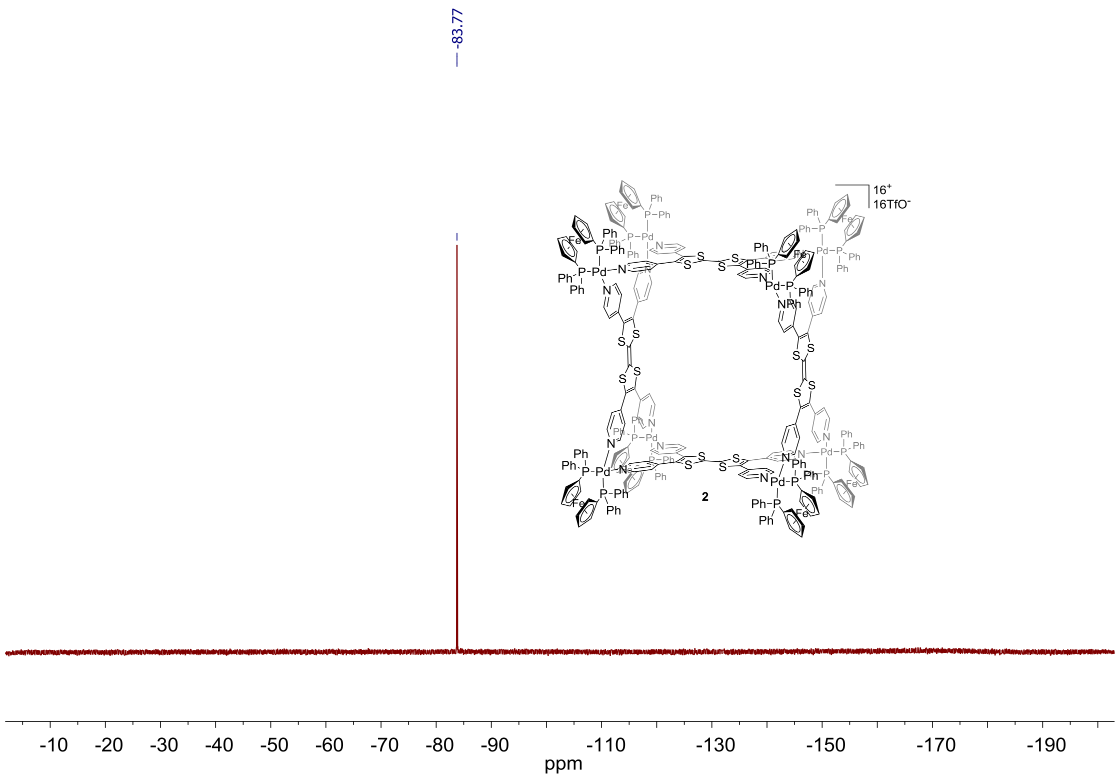

Figure S10. ^{31}P NMR spectrum of **2** in CD_3NO_2.

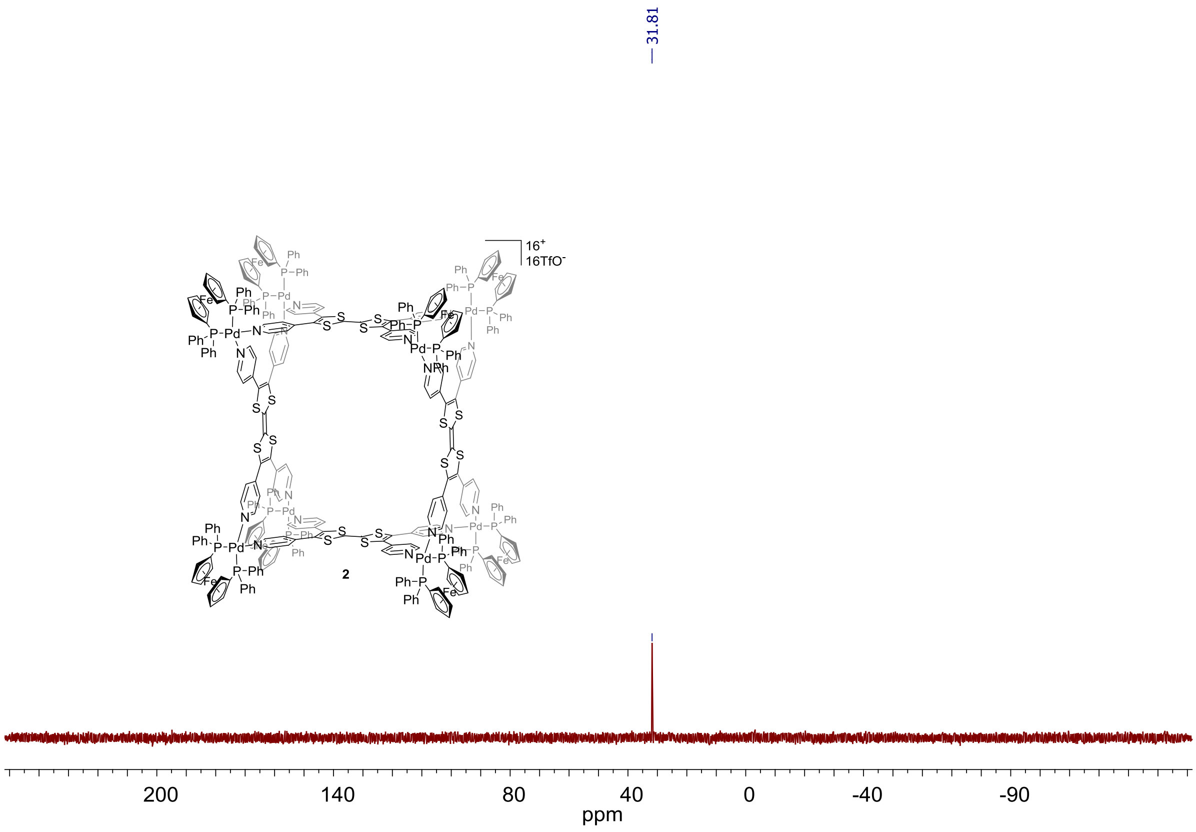

Figure S11. DOSY NMR spectrum of **2** in CD_3NO_2.

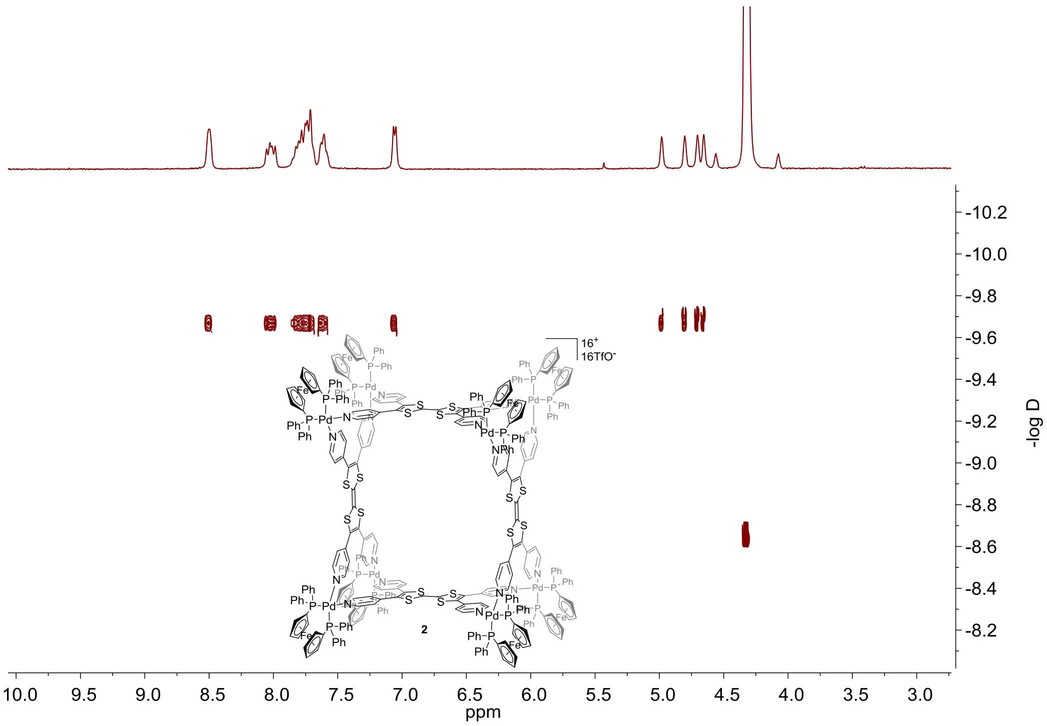

Figure S12. COSY NMR spectrum of **2** in CD_3NO_2/CD_2Cl_2 (2/1).

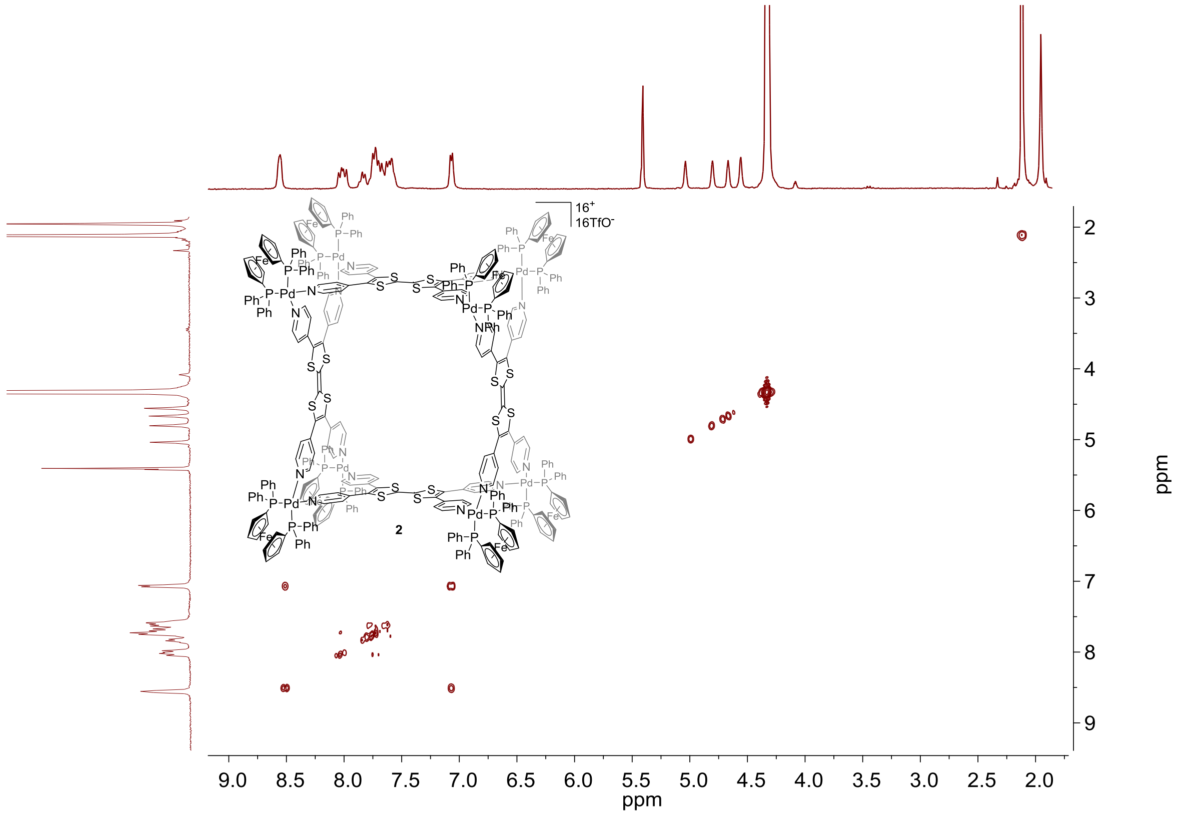

Mass Spectra of 1 and 2

Figure S13. ESI-MS spectrum of **1** in CH_2Cl_2.

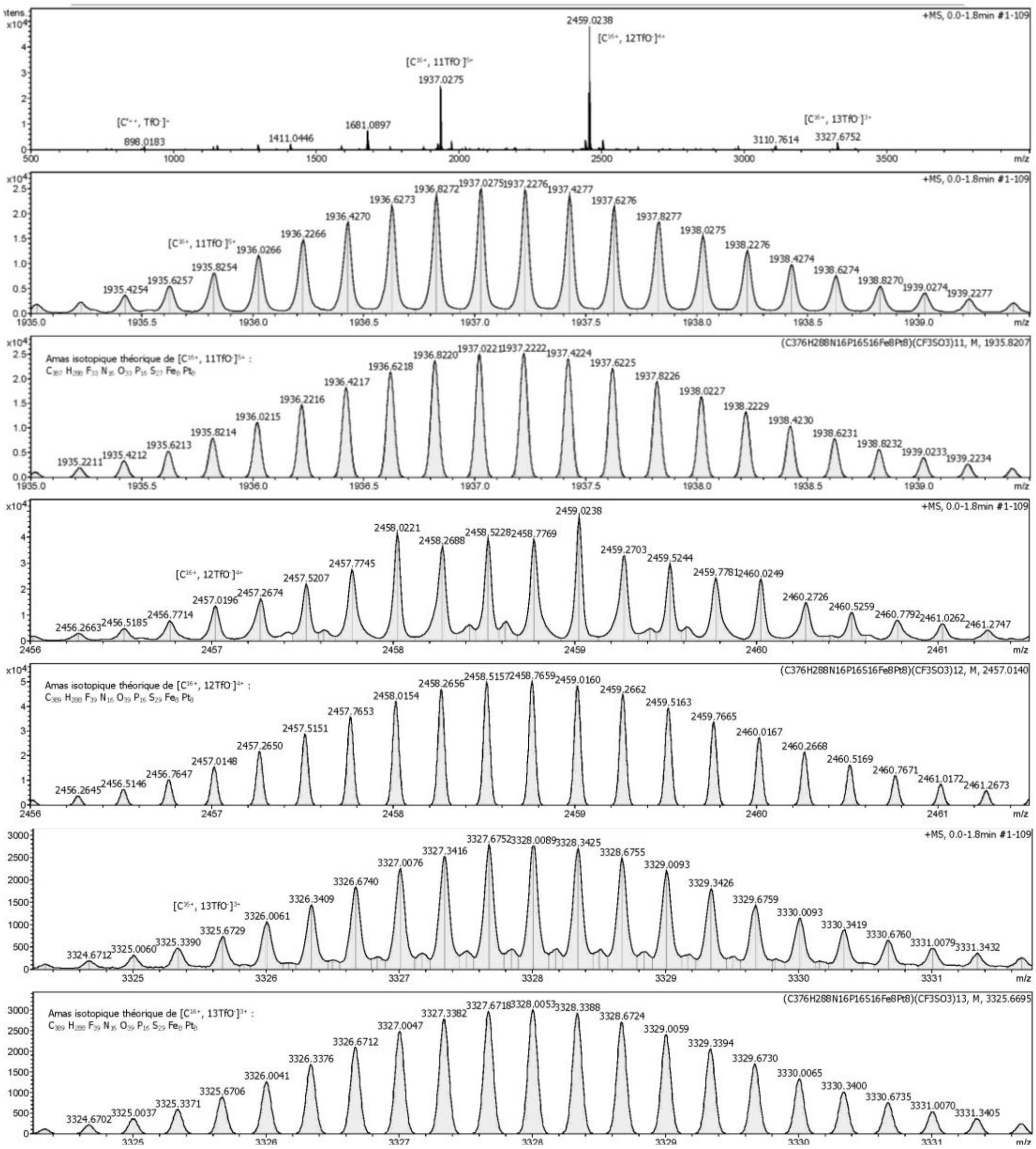

Figure S14. ESI-MS spectrum of **2** in CH_2Cl_2.

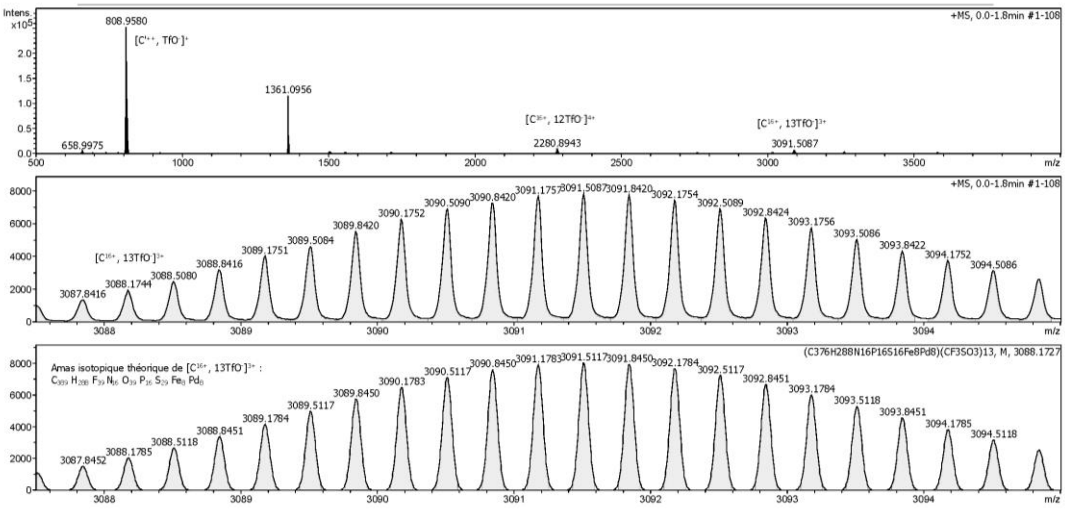

X-Ray Structures

Table S1. Crystal data and structure refinement for ligand **L1**.

Compound		**L1**
Empirical formula		$C_{26}H_{16}N_4S_4$
Crystal description and colour		red needle
Temperature (K)		180(2)
Crystal system		monoclinic
Space group		*P* 2_1/*n*
Unit cell dimensions	a (Å)	5.699(1)
	b (Å)	11.373(1)
	c (Å)	17.859(3)
	–	90
	–	94.07(2)
	–	90
Cell volume (Å^3)		1154.6(3)
Z		2
Collected / Unique reflections		26136/3337
Parameters		186
Residual factors [I > 2σ(I)]	R1	0.0438
	wR2	0.0722
	G.O.F.	1.042

Figure S15. Crystal packing of **L1** in the bc plane.

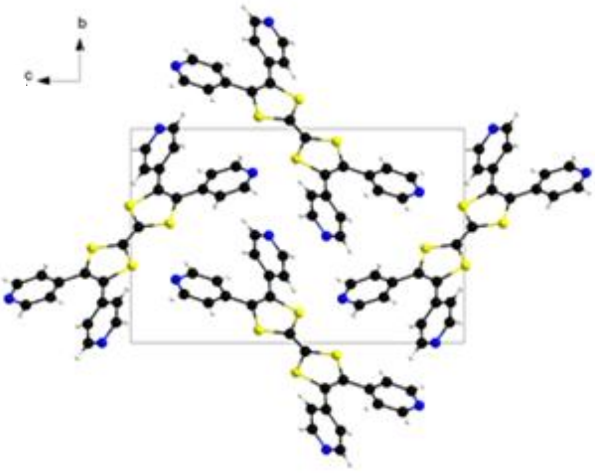

Supplement: Supplementary File 1 [file materials-07-00611-s001.pdf]
